# TRiC/CCT Complex, a Binding Partner of NS1 Protein, Supports the Replication of Zika Virus in Both Mammalians and Mosquitoes

**DOI:** 10.3390/v12050519

**Published:** 2020-05-08

**Authors:** Yuchen Wang, Ryuta Uraki, Jesse Hwang, Erol Fikrig

**Affiliations:** 1 State Key Laboratory of Virology, College of Life Science, Wuhan University, Wuhan 430072, China; 2 Section of Infectious Diseases, Department of Internal Medicine, Yale University School of Medicine, New Haven, CT 06519, USA; jesse.hwang@yale.edu (J.H.); erol.fikrig@yale.edu (E.F.); 3 Department of Immunology, Graduate School of Medical Sciences, Nagoya City University, Nagoya 467-8601, Japan; 4 Howard Hughes Medical Institute, Chevy Chase, MD 20815, USA

**Keywords:** Zika virus, NS1 protein, TRiC/CCT complex, viral replication, *Aedes aegypti* mosquito

## Abstract

Mosquito-borne Zika virus (ZIKV) can cause congenital microcephaly and Guillain–Barré syndrome, among other symptoms. Specific treatments and vaccines for ZIKV are not currently available. To further understand the host factors that support ZIKV replication, we used mass spectrometry to characterize mammalian proteins that associate with the ZIKV NS1 protein and identified the TRiC/CCT complex as an interacting partner. Furthermore, the suppression of CCT2, one of the critical components of the TRiC/CCT complex, inhibited ZIKV replication in both mammalian cells and mosquitoes. These results highlight an important role for the TRiC/CCT complex in ZIKV infection, suggesting that the TRiC/CCT complex may be a promising therapeutic target.

## 1. Introduction

Zika virus (ZIKV) is a mosquito-borne enveloped, positive-strand RNA virus in the genus *Flavivirus* and the family Flaviviridae [1]. ZIKV is primarily transmitted to mammals via the bite of an *Aedes aegypti* mosquito [2]. While symptoms are generally mild in most individuals, ZIKV infection of pregnant women can cause intrauterine growth restriction and microcephaly [3]. As no licensed vaccine or specific antiviral treatment is available, understanding the molecular mechanisms of ZIKV infection is crucial to develop countermeasures [4,5].

The flavivirus RNA genome encodes three structural (capsid, premembrane, and envelope) and seven nonstructural genes (*NS1*, *NS2A*, *NS2B*, *NS3*, *NS4A*, *NS4B*, and *NS5*), with untranslated regions (UTR) flanking the 5′ and 3′ ends [6]. ZIKV nonstructural proteins play roles in viral replication and assembly [7]. NS1 is a highly conserved nonstructural protein among the flaviviruses, which possesses multiple functions in the viral life cycle, including viral replication, immune evasion, and pathogenesis [8]. A previous study reported that an attenuated recombinant vesicular stomatitis virus-based vaccine expressing ZIKV prM-E-NS1 induces ZIKV-specific antibody and T cell immune responses, providing protection against ZIKV challenge [9]. Monoclonal antibodies targeting NS1 protein can also protect against disease and death in a murine model [10]. These studies suggest that ZIKV NS1 could be a potential therapeutic target. 

Here, we utilized liquid chromatography–tandem mass spectrometry (LC-MS/MS) to identify host factors that interact with the ZIKV NS1 protein. Among the putative interactions, we validated the oligomeric chaperonin containing TRiC (TCP-1 ring complex), as NS1-binding host factor. The TRiC/CCT complex, consisting of eight different subunits (CCT1–CCT8), is essential for protein folding in an ATP-dependent manner [11], and previous studies showed that the TRiC/CCT complex participates in the life cycle of several viruses, including DENV [12], hepatitis C virus [13], and reovirus [14]. We corroborate the functional role of TRiC/CCT complex during ZIKV replication by showing that CCT2 suppression reduces ZIKV replication in not only human cells but also in mosquitoes. These results demonstrate an important role of TRiC/CCT complex for the ZIKV life cycle in mammalian hosts and arthropod vectors and suggest that targeting this host factor may offer protection against ZIKV pathogenesis.

## 2. Materials and Methods

### 2.1. Ethics Statement

All experiments were performed in accordance with guidelines from the Guide for the Care and Use of Laboratory Animals (National Institutes of Health). The animal experimental protocols were approved by the Institutional Animal Care and Use Committee (IACUC) at the Yale University School of Medicine (assurance number A3230-01). The Assurance was approved 5 May 2015. All infection experiments were performed in a biosafety level 2 and arthropod containment level 3 lab (ACL3) animal facility according to the regulations of Yale University.

### 2.2. Viruses, Cell Lines, Mosquitoes, and Antibodies

An Asian-derived Cambodian strain (FSS13025 strain, referred to ZIKV^Cam^, obtained from The University of Texas Medical Branch, Galveston, TX, USA) isolated in 2010 and Mexican strain (MEX2-81 strain, ZIKV^Mex^, obtained from The University of Texas Medical Branch, Galveston, TX, USA) isolated in 2016 were used for infection. HEK293T cells (human embryonic kidney, ATCC, Manassas, VA, USA) and Hela cells (human cervical cancer, ATCC, Manassas, VA, USA) were used for vitro studies. *Aedes aegypti* (Orlando strain, obtained from the Connecticut Agricultural Experiment Station, New Haven, CT, USA) mosquitoes were used for vivo studies. The rabbit anti-human CCT2 (One World Lab, San Diego, CA, USA), rabbit anti-ZIKV NS1 (Genetex, Irvine, CA, USA), rabbit anti-ZIKV Capsid (Cap) (Genetex, Irvine, CA, USA), mouse anti-HA (Abcam), mouse anti-c-Myc (Sigma-Aldrich, Burlington, MA, USA), mouse anti-actin (Abcam, Cambridge, MA, USA), HRP-linked rat anti-mouse IgG (Mouse TrueBlot^®^ ULTRA, ROCKLAND, Limerick, PA, USA), HRP-linked goat anti-rabbit IgG (Cell Signaling, Danvers, MA, USA), mouse anti-ZIKV NS1 monoclonal (GeneTex, Irvine, CA, USA), rabbit anti-CCT2 monoclonal (Abcam, Cambridge, MA, USA), goat anti-mouse IgG (H+L) cross-adsorbed secondary antibody-Alexa Fluor 555 (Invitrogen, Carlsbad, CA, USA), and F(ab’)2-goat anti-rabbit IgG (H+L) cross-adsorbed secondary antibody-Alexa Fluor 488 (Invitrogen, Carlsbad, CA, USA) antibodies were purchased. Pierce™ Anti-HA Magnetic Beads and Pierce™ Anti-c-Myc Magnetic Beads were purchased from Thermo Fisher (Branchburg, NJ, USA).

### 2.3. Pull-Down Assay and Mass Spectrometry

The ORF of NS1 from ZIKV^Cam^ was cloned into plasmid pcDNA4.1 in-frame with a c-Myc-His-tag sequence for the expression of C-terminally c-Myc-His-tagged NS1, c-Myc-His-tagged NS1 mutants, or naïve NS1 (no-tag). TRiC/CCT complex gene was cloned into plasmid pcDNA4.1 in-frame with a HA-tag sequence. 293T cells were transfected with plasmid DNA encoding HA-CCT1-8, naïve NS1, NS1-c-Myc-His, and NS1 deletion mutants by TransIT 2020 (Mirus, Madison, WI, USA). After 24 h post transfection, 293T cells were lysed with lysis buffer (50 mM Tris-HCl, 150 mM NaCl, 0.5% NP-40, and protease inhibitor cocktail). The supernatants were incubated with anti-c-Myc magnetic beads according to manufacturer’s protocol. Immunoprecipitated proteins were eluted with Laemmli sample buffer (Biorad, Portland, ME, USA) and then subjected to SDS-PAGE followed by silver staining (Thermo Fisher kit, Branchburg, NJ, USA). Protein bands after silver staining were excised from the gel and were analyzed at the Yale University W.M. Keck Foundation core facility (New Haven, CT, USA). The samples were subjected to trypsin digestion followed by LC-MS/MS for peptide sequencing and identification. 

### 2.4. Immunoprecipitation and Immunoblotting

HEK 293T cells were transfected with the plasmids by using the TransIT. After 24 h post transfection, cells were lysed as described above. The supernatants were incubated with anti-HA or anti-c-Myc magnetic beads according to manufacturer’s protocol. To examine the effect of ATP on the interaction, several concentrations of ATP (0, 10, 50, 100 mM) and 10 mM MgCl_2_ were added in Lysis buffer described above. Proteins bound to the beads were harvested and separated by SDS-PAGE. Proteins were transferred onto polyvinylidene difluoride (PVDF) membranes (Millipore, Bedford, MA, USA). The blots were blocked in 1% non-fat milk. Primary antibodies and horseradish peroxidase-conjugated secondary antibodies were diluted and incubated with the blots. After washing with 0.05% PBS-T, the immunoblots were imaged through chemiluminescent reagent (GE Healthcare, Chicago, IL, USA) with a LI-COR Odyssey imaging system (LI-COR, Lincoln, NE, USA).

### 2.5. Immunofluorescence Assay and Confocal Microscopy

HeLa cells were washed with phosphate-buffered saline (PBS) at 48 h ZIKV^Cam^ post infection, followed by fixing in 4% (*vol*/*vol*) paraformaldehyde (Electron Microscopy Sciences, Hatfield, PA, USA) for 15 min at room temperature, and permeabilized with 0.1% Triton X-100 (Sigma, Burlington, MA, USA). The cells were blocked with 3% (*wt*/*vol*) BSA and 5% non-fat dry milk in PBS for 1 h. The cells were subsequently immunolabeled with a mixture of primary antibodies for 1 h at room temperature at the following dilutions: the mouse anti-ZIKV NS1 monoclonal (GeneTex, Irvine, CA, USA), 1:500, the rabbit anti-CCT2 monoclonal (Abcam, Cambridge, MA, USA), 1:250. Cells were washed with PBS several times and then immunolabeled with a mixture of secondary antibodies for 1 h at room temperature at the following dilutions: Alexa Fluor 488 F(ab’)2-goat anti-rabbit IgG (H+L) cross-adsorbed secondary antibody (Invitrogen, Carlsbad, CA, USA), 1:1000, Alexa Fluor 555 goat anti-mouse IgG (H+L) cross-adsorbed secondary antibody (Invitrogen, Carlsbad, CA, USA), 1:1000 for 1 h. Nuclei were stained with 2-(4-amidinophenyl)-6-indolecarbamidine dihydrochloride (DAPI) (Sigma, Burlington, MA, USA), 1:5000 for 15 min. After staining, the fluorescence signals were examined with a laser scanning confocal microscope (TCS SP5, Leica, Wetzlar, Germany).

### 2.6. Generation of Stable Cell Lines Suppressing CCT2

To generate cell lines that stably suppress CCT2 protein, Hela cells were transfected with CCT2 MISSION shRNA Plasmid (Thermo Fisher, Branchburg, NJ, USA). Cell clones were selected in DMEM supplemented with 10% (*vol*/*vol*) fetal calf serum (FCS), penicillin G (100,000 U/L), streptomycin (100 mg/L), and puromycin (2 μg/mL). Immunoblot was performed to confirm that the expression of CCT2 was suppressed in a selected clone.

### 2.7. Treatment of HSF1A and LDH Cytotoxicity Assay

HeLa cells were treated with HSF1A (0, 25, 50, 75, or 100 µM) 12 h prior to Zika virus infection. After Zika virus infection, infected cells were cultured in the media containing HSF1A (0, 25, 50, 75, or 100 µM). At several timepoints post infection (0, 12, 24, and 36 h), supernatants or whole-cell lysates were collected and analyzed by plaque assay or immunoblotting. The cytotoxicity of HSF1A toward HeLa cells was measured using a Pierce LDH Cytotoxicity Assay Kit (Thermo Scientific, Branchburg, NJ, USA). HeLa cells seeded into 48-well plates were treated with HSF1A compound at 0, 25, 50, 75, 100 μM HSF1A and DMSO (Negative control) for 48 h. Lactate dehydrogenase (LDH) released in the supernatants was measured as a cytotoxicity parameter.

### 2.8. Gene Silencing in Mosquitoes

Double-stranded (ds) RNA targeting either a 400 bp region of the *A. aegypti CCT2* gene or an irrelevant green fluorescent protein (GFP) gene were transcribed using gene-specific primers designed with a T7 promoter and the MEGAScript RNAi kit (Thermo Fisher Scientific, Ambion, Branchburg, NJ, USA). DsRNA was produced using TranscriptAid T7 High Yield Transcription Kit (ThermoFisher, Branchburg, NJ, USA) and purified using phenol-chloroform extraction and ethanolprecipitation. Adult female *A. aegypti* mosquitoes were injected with 500 ng dsRNA in PBS into the thorax to silence the *A. aegypti CCT2* gene. At day 3 following dsRNA injection, the mosquitoes were injected with ZIKV^Mex^ (100 PFU). At day 3, 7 days following virus injection, the whole mosquitoes were collected to examine the *CCT2* expression levels by qRT-PCR to make sure the efficacy of dsRNA against *CCT2* was as described below.

### 2.9. Quantitative Real-Time PCR

RNA from cells was extracted with RNeasy mini kit (Qiagen, Germantown, MD, USA), and the mosquito total RNA was extracted using TRIzol reagent (Ambion by Life Technologies, Branchburg, NJ, USA) according to manufacturer’s protocol. DsRNA was produced using TranscriptAid T7 High Yield Transcription Kit (ThermoFisher, Branchburg, NJ, USA) and purified using phenol-chloroform extraction and isopropanol precipitation. The cDNA was generated with an iScript cDNA synthesis kit (Bio-Rad, Portland, ME, USA) according to manufacturer’s protocol. Gene expression was examined by qRT-PCR using IQ SYBR Green Supermix. Viral RNA or Cct2 RNA levels were normalized to mosquito RP49 RNA levels according to 2^−ΔΔCt^ calculations.

### 2.10. Statistical Analysis

GraphPad Prism software (GraphPad, San Diego, CA, USA) was used to perform statistical analysis on all data. Mosquitoes were randomly allocated into different groups. No statistical methods were used to predetermine sample size. The viral titers were analyzed using two-way ANOVA with Bonferroni’s multiple comparisons test or one-way ANOVA with Dunnett’s multiple comparisons test. Rp49-normalized viral RNA levels were analyzed using the Wilcoxon–Mann–Whitney test. A *p*-value less than 0.05 was considered statistically significant, and all significant *p*-values are listed in the Figure legends.

### 2.11. Data Availability

Data that support the findings of this study are available from the corresponding authors upon request.

## 3. Results

### 3.1. TRiC/CCT Complex Interacts with the Zika Virus NS1 Protein

**Figure 1:**
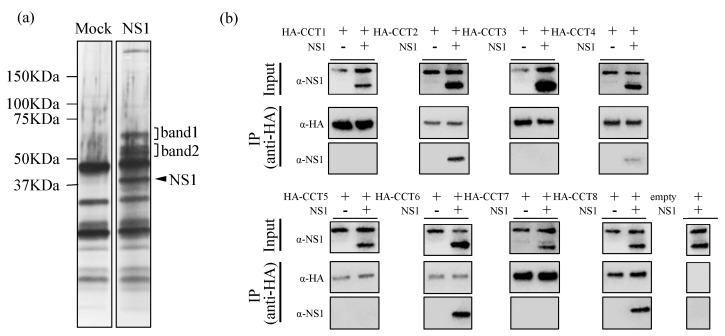
Identification of the interaction of the human 293T cell proteins with Zika virus NS1 protein. (**a**) The identification of host factors binding to Zika virus NS1. Human 293T cells were transfected with c-Myc-tagged NS1-expressing vector. Twenty-four hours post transfection, the whole mock and NS1-transfected cell lysates were incubated with anti-c-Myc magnetic beads, then analyzed by SDS-PAGE, followed by staining with silver staining. The arrowheads indicate the position of NS1 protein and the band 1 and band 2 which are interacted with NS1 protein. (**b**) Co-immunoprecipitation of NS1 with CCT1-8. HA-tagged CCT1-8 proteins and naïve (nontagged) NS1 protein were expressed in 293T cells. After immunoprecipitation with anti-HA beads, samples were analyzed by SDS-PAGE, followed by immunoblotting using a HA tag or NS1-specific antibody.

To identify host proteins that interact with the Zika virus NS1 protein, we transfected 293T cells with a plasmid expressing Zika virus *NS1* gene (corresponding to amino acid residues 1-352) with c-Myc tag at C-terminus. After removal of cellular debris, the supernatants from mock and NS1-transfected cells were incubated with anti-c-Myc magnetic beads then analyzed by SDS-PAGE, followed by silver staining (Figure 1a). Two unique regions of stained gel were subjected to in-gel trypsin digestion for protein identification, followed by LC-MS/MS. The unique identities of band 1 and band 2 are shown in Table 1. Interestingly, all subunits of the eight-subunit chaperonin containing TCP-1 (TRiC/CCT complex), whose theoretical masses of the identified proteins were compatible with their locations on the gel, were identified as binding partners to NS1 protein.

TRiC/CCT complex is a molecular chaperone belonging to the family of chaperonins, a conserved class of large double-ring complex of 800 kDa enclosing a central cavity, and is found in the cytoplasm of all eukaryotic cells [15,16]. It is composed of two identical rings, each composed of eight different subunits (CCT1–CCT8), and mediates cytosolic protein folding and assembly [17,18,19]. In order to examine the specificity of the interaction of Zika virus NS1 protein with subunits of TRiC/CCT complex, we cotransfected expression plasmids encoding HA-tagged CCT1-8 and nontagged NS1 protein, and performed coimmunoprecipitation assay. We found that CCT2, CCT4, CCT6, and CCT8 interact with NS1 protein (Figure 1b), suggesting NS1 protein uses the TRiC/CCT complex as a chaperone during ZIKV replication.

### 3.2. CCT2 Interacts with the Central Region of Zika Virus NS1

**Figure 2:**
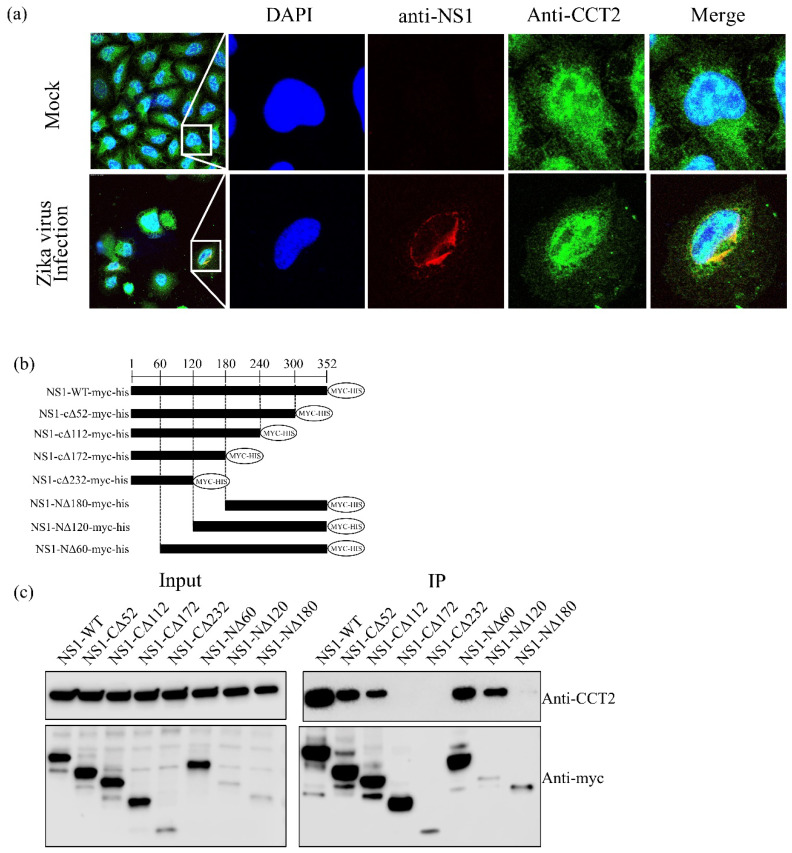
Identification of the Zika virus NS1 region interacting with CCT2 in mammalian cells. (**a**) HeLa cells were infected with ZIKV^Cam^, and colocalization of ZIKV NS1 and CCT2 were examined using confocal microscopy (Leica SP5, 100× objective): blue, cell nucleus; red, ZIKV NS1 protein; green, CCT2 protein. Right panels show enlargement of area indicated by the white boxes. (**b**) Diagrams of NS1 deletion mutants. A series of NS1 deletion mutants was constructed by sequential deletion from the N- or C-terminus of NS1. A c-Myc-tag was added at the C-terminus. (**c**) Immunoprecipitation of wild-type and deletion mutant NS1 proteins with naïve CCT2. Wild-type and the indicated deletion mutant NS1 proteins were expressed in 293T cells. Twenty-four post transfection, immunoprecipitations were performed with anti-c-Myc beads, followed by immunoblotting.

As CCT2 (T-complex protein 1 subunit beta) was confirmed to bind NS1 protein by immunoprecipitation, and the Mascot score, a statistical score for how well the experimental data match the database protein sequences, was highest among the eight subunits of TRiC/CCT complex, we further investigated the contribution of CCT2 to ZIKV replication. First, the colocalization of the ZIKV NS1 with CCT2 was examined by confocal microscopy (Figure 2a), suggesting that ZIKV NS1 and CCT2 were colocalized in the cytoplasm of HeLa cells. To determine the regions responsible for the interaction, we generated a series of NS1 deletion mutants with c-Myc-His tag at the C-termini of NS1 (Figure 2b), co-expressed them with CCT2, and immunoprecipitated with anti-c-Myc magnetic beads. The expression levels of NS1 deletion mutants and CCT2 were confirmed by immunoblotting with anti-c-Myc and anti-CCT2 antibodies, respectively (Figure 2c). Although the binding affinity differed in strength, NS1 deletion mutants NS1-CΔ52, NS1-CΔ112, NS1-NΔ60, and NS1-NΔ120 coimmunoprecipitated with CCT2. By contrast, NS1-NΔ180 rarely bound with CCT2 and two NS1 deletion mutants (NS1-CΔ172 and NS1-CΔ232) failed to bind with CCT2. These data suggest that the region spanning amino acids at position 121–240 of NS1 is responsible for interaction with CCT2.

### 3.3. Interaction of ZIKV NS1 with CCT2 Depends on ATP Concentration

**Figure 3:**
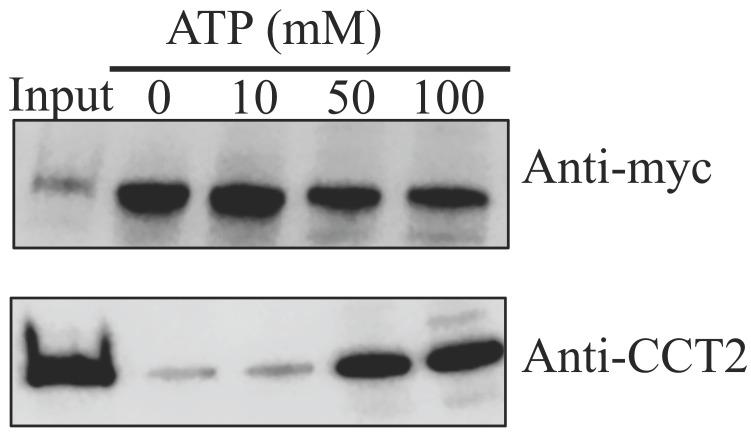
ATP-dependent interaction between CCT2 and Zika virus NS1. c-Myc tagged NS1 was expressed in human 293T cells. Twenty-four post transfection, immunoprecipitation was performed with anti-c-Myc beads under several ATP concentrations (0, 10, 50, 100 mM), followed by immunoblotting.

The central chamber of the TRiC/CCT complex folds many essential cellular proteins in an ATP-dependent manner [11]. As we did not add any ATP to the buffer in our previous immunoprecipitation system, we examined the effect of ATP concentration on the interaction of NS1 with CCT2. Immunoprecipitation was performed with anti-c-Myc beads under several concentrations of ATP (0, 10, 50, 100 mM). We found that the interaction between CCT2 and NS1 was ATP-dependent (Figure 3), suggesting ATP concentration may modulate the binding between ZIKV NS1 and TRiC/CCT complex, contributing to the ZIKV replication.

### 3.4. Knockdown of CCT2 Reduces Zika Virus Replication in Mammalian Cells

**Figure 4:**
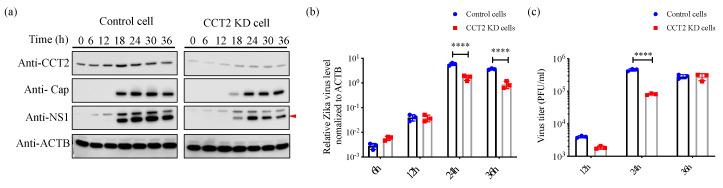
The effect of suppression of CCT2 on Zika virus replication. (**a**) CCT2 knockdown or control HeLa cells were infected with ZIKV at Multiplicity of Infection (MOI) of 1. At the indicated timepoints post infection (0, 6, 12, 18, 24, 30, and 36 h), samples were harvested and analyzed by immunoblotting with indicated antibodies. β-actin (ACTB) was used as a loading control. Red arrow shows detected NS1 protein. (**b**) CCT2 knockdown or control HeLa cells were infected with ZIKV at MOI of 1. At the indicated timepoints post transfection (6, 12, 24 and 36 h), samples were harvested and viral RNA levels were analyzed by qRT-PCR. Viral RNA levels were normalized to β-actin. (**c**) CCT2 knockdown and control cells were infected with ZIKV at MOI of 0.05. At the indicated timepoints post infection (12, 24, and 36 h), supernatants were collected, and virus titers were determined by plaque assay in Vero cells. **** *p* < 0.0001 by two-way ANOVA with multiple comparisons test. Data are plotted as the mean ± SEM.

To examine the biological function of CCT2 during the ZIKV life cycle, we generated shRNA-mediated knockdown (KD) of CCT2 in HeLa cells and assayed for virus replication. An shRNA targeting GFP was used as a negative control. At every 6 h after infection with ZIKV^Cam^, we harvested samples for testing protein expression and found that the expression levels of viral proteins and RNA were suppressed or delayed in CCT2-KD cells compared with control cells (Figure 4a,b). To investigate the effect of CCT2 knockdown on the production of infectious particles, we infected KD and control cells with ZIKV^Cam^. As shown in Figure 4c, downregulation of CCT2 expression significantly reduced the replication of ZIKV. These results suggest that TRiC/CCT complex supports ZIKV life cycle.

### 3.5. Inhibition of TRiC/CCT Complex Function Using HSF1A Reduces Zika Virus Propagation

**Figure 5:**
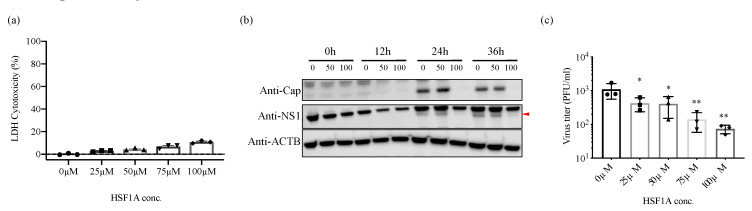
The effect of TRiC/CCT inhibitor HSF1A on ZIKV propagation. (**a**) HeLa cells treated with HSF1A (0, 25, 50, 75, or 100 µM) or DMSO (control) for 48 h, after which lactate dehydrogenase (LDH) released in the supernatants was assayed. (**b**) HeLa cells were pretreated with HSF1A (0, 50, or 100 µM) for 12 h and infected with ZIKV at MOI of 1. Infected cells were maintained in media containing HSF1A (0, 50, or 100 µM). At the indicated timepoints post infection (0, 12, 24, and 36 h), whole-cell lysates were harvested and analyzed by immunoblotting with indicated antibodies. β-actin was used as a loading control. Red arrow shows detected NS1 protein. (**c**) HeLa cells were pretreated with HSF1A (0, 25, 50, 75, or 100 µM) for 12 h and infected with ZIKV at MOI of 0.1. Supernatants were harvested and titrated at 36 h post infection by plaque assay. * *p* < 0.05 and ** *p* < 0.01 by one-way ANOVA with Dunnet’s multiple comparisons test. Data are plotted as the mean ± SEM.

HSF1A is a cell-permeable compound that inhibits the function of TRiC/CCT [20]. To further elucidate the importance of TRiC/CCT complex during the ZIKV replication cycle, HeLa cells were treated with 0, 50, and 100 μM of HSF1A 12 h prior to ZIKV infection. The expression of viral proteins was suppressed in 50 and 100 μM HSF1A-treated cells compared with control cells with little toxicity (Figure 5a,b). The expression level of β-actin, whose expression/conformation is supported by TRiC/CCT complex, was not altered after HSF1A treatment, suggesting that the toxicity of HSF1A unlikely affects the expression level of viral proteins. To better understand the effect of HSF1A on the production of infectious particles, HeLa cells were treated with HSF1A 12 h prior to ZIKV infection. The amount of infectious virus in the supernatant was determined at 36 h post infection. Notably, ZIKV replication was significantly reduced in HeLa cells in a dose-dependent manner with little toxicity (Figure 5a,c). These findings support TRiC/CCT complex having an important role in the ZIKV replication cycle.

### 3.6. Suppression of CCT2 Reduces Zika Virus Burden in Mosquitoes

**Figure 6:**
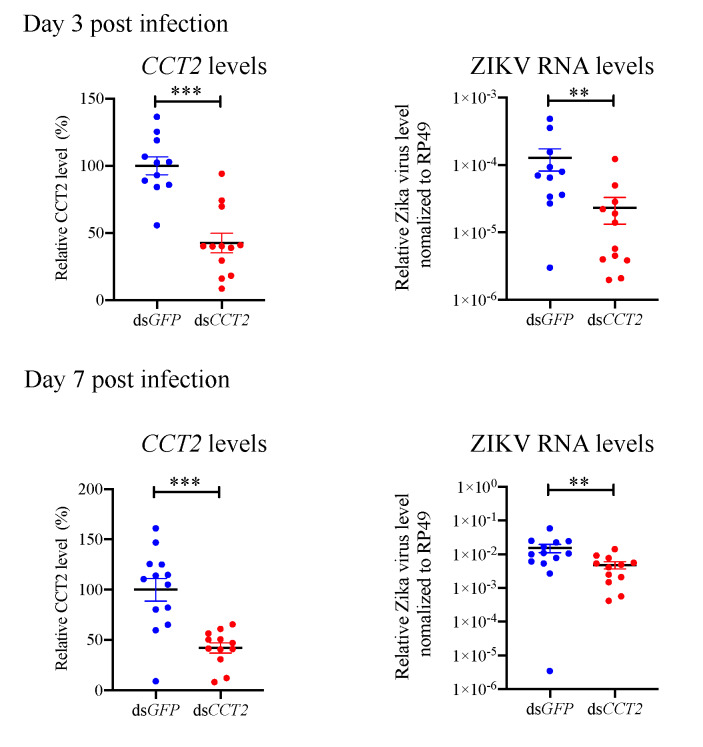
The effect of suppression of CCT2 in *Aedes aegypti* mosquitoes on Zika virus replication. *Aedes aegypti* mosquitoes were intrathoracically injected with 500 ng dsRNA targeting *green fluorescent protein (GFP*) (control) or *CCT2* gene. After 3 days post dsRNA injection, *Aedes aegypti* mosquitoes were injected with 100 PFU of ZIKV. (**left panels**) At 3 and 7days post ZIKV injection, *CCT2* expression levels in the whole *Aedes aegypti* mosquito were analyzed by qRT-PCR (Day 3: ds*GFP*-treated mosquitoes: *n* = 11, ds*CCT2*-treated mosquitoes: *n* = 12, Day 7: ds*GFP*-treated mosquitoes: *n* = 13, ds*CCT2*-treated mosquitoes: *n* = 12). CCT2 RNA levels were normalized to the levels of *Rp49*. (**right panels**) At 3 and 7 days post ZIKV injection, viral RNA levels in the whole *Aedes aegypti* mosquito were analyzed by qRT-PCR (Day 3: ds*GFP*-treated mosquitoes: *n* = 11, ds*CCT2*-treated mosquitoes: *n* = 12, Day 7: ds*GFP*-treated mosquitoes: *n* = 13, ds*CCT2*-treated mosquitoes: *n* = 12). Viral RNA levels were normalized to the levels of *Rp49*. Data are representative of three independent experiments with similar results. Significance is shown with asterisk ** *p* < 0.01 and *** *p* < 0.005 by Wilcoxon–Mann–Whitney test. Data are presented as the mean ± SEM.

TRiC/CCT complex is conserved in all eukaryotic cells [21,22,23]. Thus, we hypothesized that this complex may regulate ZIKV infection in mosquitoes, which are vectors for ZIKV transmission cycle [24,25,26,27]. In order to investigate the role of this complex in mosquitoes, we silenced the *CCT2* gene in *A. aegypti* mosquitoes using RNAi, and then investigated whether suppression of *CCT2* gene expression alters ZIKV replication in mosquitoes. After 3 days post dsRNA injection, *A. aegypti* mosquitoes were injected with 100 PFU of ZIKV^Mex^. Interestingly, there was a significant reduction in the levels of ZIKV in the *CCT2* dsRNA-treated mosquitoes compared with the control *GFP* dsRNA-treated mosquitoes (Figure 6). This result demonstrates that the TRiC/CCT complex contributes to ZIKV replication in mosquitoes as well as mammalian hosts.

## 4. Discussion

Various screening methods for flavivirus–host protein interactions have been used to study the pathogenesis of flavivirus [12,28,29]. NS1 is a nonstructural protein highly conserved among the flaviviruses [30] and possesses multiple functions in the viral life cycle, including viral replication, immune evasion, and pathogenesis [8]. A previous study reported that an attenuated recombinant vesicular stomatitis virus (rVSV)-based vaccine expressing ZIKV prM-E-NS1 could induce Zika virus-specific antibody and T cell immune responses, thus providing protection against ZIKV challenge [9]. Also, mAbs targeting NS1 protein can protect against disease and death in a murine model [10]. These studies suggest that ZIKV NS1 could be a potential therapeutic target.

In this study, we identify the TRiC/CCT complex as a binding partner of the NS1 protein by pull-down assay followed by LC-MS/MS (Table 1). TRiC/CCT complex is a type II chaperonin composed of eight different subunits (CCT1–CCT8) that assists in the folding and assembly of essential host cytosolic proteins [31,32] and has been shown to be required for replication of several viruses [13,14,33,34,35], including flaviviruses [12,36,37]. Here, we found that several subunits (CCT2, CCT4, CCT6A, and CCT8) of TRiC/CCT complex are able to bind ZIKV NS1 (Figure 1b), suggesting that NS1 protein uses the TRiC/CCT complex as a chaperone during ZIKV replication.

Our data demonstrated that the region spanning amino acids at position 121–240 of NS1 is responsible for interaction with CCT2 (Figure 2). Several studies reporting the crystal structures of NS1 indicate that amino acids 121–240 might be involved in interactions with structural proteins Envelope (E) and precursor Membrane (prM) [38,39]. Interestingly, patients infected with Dengue virus (DENV) induce antibodies recognizing the region between amino acids 221 and 266 of DENV NS1, which is highly conserved among DENV serotypes [40]. Another study also demonstrated that the region located between amino acids 225 and 245 was highly conserved, which is beneficial for DENV vaccine design [41]. In addition, several monoclonal antibodies against WNV NS1 recognize amino acids 158 to 235, leading to strong protection against WNV in mice [42]. Considering the high structural similarity of NS1 among flaviviruses, targeting the 121–240 domain of the ZIKV NS1 could not only perturb NS1 function but also influence the interaction between the TRiC/CCT complex and NS1. Therefore, analyzing the spatiotemporal dynamics of NS1–CCT binding would be helpful to dissect this facet of host–pathogen interaction with greater precision.

Previous studies showed that the TRiC/CCT complex participates in the replication of several viruses, including DENV [12], hepatitis C virus [13], and reovirus [14]. Here, we found that the expression levels of viral proteins and RNA and the production of infectious particles were suppressed or delayed in CCT2-KD cells and in TRiC/CCT inhibitor-treated cells compared with control cells (Figure 4 and Figure 5), suggesting that TRiC/CCT complex can be a therapeutic target against multiple viruses.

Interestingly, our result demonstrates that the TRiC/CCT complex contributes to ZIKV replication in mosquito vectors as well (Figure 6). These data suggest the possible prevention of ZIKV spread in mosquitoes by using compounds targeting TRiC/CCT complex.

In conclusion, our study identified the TRiC/CCT complex as a binding partner of ZIKV NS1. Silencing the function of this complex reduces viral burden in mammalian cells and mosquitoes. These results highlight an important role for the relationship between NS1 and the TRiC/CCT complex during ZIKV infection. Although we need to investigate a possibility that viral RNA reduction in the CCT2 knockdown mosquitoes or viral reduction in the CCT2 knockdown cells is caused by diminished cell metabolism, we did not observe overt toxicity. Our results suggest that TRiC/CCT complex is exploited by ZIKV for maximal replication in both mammals and arthropod vectors and that TRIC/CCT complex may be a promising target for intervention.

## Figures and Tables

**Table 1:** Identification of Zika virus (ZIKV) NS1 protein-binding partners by LC-MS/MS (Liquid chromatography–mass spectrometry).

Protein ID	Protein Name	Mass (Da)	Sequence Coverage (%)	Mascot Score
Band 1				
GRP78_HUMAN	78 kDa glucose-regulated protein Organism Name (OS) = Homo sapiens Gene Name (GN) = HSPA5 Protein Existence (PE) = 1 Sequence Version (SV) = 2	72,288	6.4	225
ZG16B_HUMAN	Zymogen granule protein 16 homolog B OS = Homo sapiens GN = ZG16B PE = 1 SV = 3	22,725	26	181
RORB_HUMAN	Nuclear receptor ROR-beta OS = Homo sapiens GN = RORB PE = 1 SV = 3	53,186	6	32
WFKN2_HUMAN	WAP, Kazal, immunoglobulin, Kunitz and NTR domain-containing protein 2 OS = Homo sapiens GN = WFIKKN2 PE = 1 SV = 1	63,898	5.2	31
COJA1_HUMAN	Collagen alpha-1(XIX) chain OS = Homo sapiens GN = COL19A1 PE = 1 SV = 3	115,149	6.5	29
ARHGC_HUMAN	Rho guanine nucleotide exchange factor 12 OS = Homo sapiens GN = ARHGEF12 PE = 1 SV = 1	173,125	3.5	29
ZN469_HUMAN	Zinc finger protein 469 OS = Homo sapiens GN = ZNF469 PE = 2 SV = 3	409,949	1.1	26
KNG1_HUMAN	Kininogen-1 OS = Homo sapiens GN = KNG1 PE = 1 SV = 2	71,912	5.7	26
EEA1_HUMAN	Early endosome antigen 1 OS = Homo sapiens GN = EEA1 PE = 1 SV = 2	162,367	3.9	26
ATR_HUMAN	Serine/threonine-protein kinase ATR OS = Homo sapiens GN = ATR PE = 1 SV = 3	301,172	1.6	26
CIP1_HUMAN	E3 ubiquitin-protein ligase CCNB1IP1 OS = Homo sapiens GN = CCNB1IP1 PE = 1 SV = 1	31,524	2.9	25
ARSJ_HUMAN	Arylsulfatase J OS = Homo sapiens GN = ARSJ PE = 2 SV = 1	67,193	1.5	23
CC180_HUMAN	Coiled-coil domain-containing protein 180 OS = Homo sapiens GN = CCDC180 PE = 2 SV = 2	190,979	0.9	22
KI2S1_HUMAN	Killer cell immunoglobulin-like receptor 2DS1 OS = Homo sapiens GN = KIR2DS1 PE = 2 SV = 1	33,624	5.6	20
Band 2				
TCPB_HUMAN	T-complex protein 1 subunit beta OS = Homo sapiens GN = CCT2 PE = 1 SV = 4	57,452	47.9	1097
TCPQ_HUMAN	T-complex protein 1 subunit theta OS = Homo sapiens GN = CCT8 PE = 1 SV = 4	59,583	35.8	665
TCPH_HUMAN	T-complex protein 1 subunit eta OS = Homo sapiens GN = CCT7 PE = 1 SV = 2	59,329	32.2	627
TCPA_HUMAN	T-complex protein 1 subunit alpha OS = Homo sapiens GN = TCP1 PE = 1 SV = 1	60,306	36.5	623
TCPZ_HUMAN	T-complex protein 1 subunit zeta OS = Homo sapiens GN = CCT6A PE = 1 SV = 3	57,988	36	530
TCPG_HUMAN	T-complex protein 1 subunit gamma OS = Homo sapiens GN = CCT3 PE = 1 SV = 4	60,495	33.2	476
TCPE_HUMAN	T-complex protein 1 subunit epsilon OS = Homo sapiens GN = CCT5 PE = 1 SV = 1	59,633	30.9	458
TCPD_HUMAN	T-complex protein 1 subunit delta OS = Homo sapiens GN = CCT4 PE = 1 SV = 4	57,888	18	360
VIME_HUMAN	Vimentin OS=Homo sapiens GN = VIM PE = 1 SV = 4	53,619	12.2	97
S10AE_HUMAN	Protein S100-A14 OS = Homo sapiens GN = S100A14 PE = 1 SV = 1	11,655	14.4	95
CH60_HUMAN	60 kDa heat shock protein, mitochondrial OS = Homo sapiens GN = HSPD1 PE = 1 SV = 2	61,016	3.3	59
KPYM_HUMAN	Pyruvate kinase PKM OS = Homo sapiens GN = PKM PE = 1 SV = 4	57,900	5.1	45
PRDX2_HUMAN	Peroxiredoxin-2 OS = Homo sapiens GN = PRDX2 PE = 1 SV = 5	21,878	10.1	43
ACTA_HUMAN	Actin, aortic smooth muscle OS = Homo sapiens GN = ACTA2 PE = 1 SV = 1 indistinguishable	41,982	4.2	39
TBA1A_HUMAN	Tubulin alpha-1A chain OS = Homo sapiens GN = TUBA1A PE = 1 SV = 1 indistinguishable	50,104	4	39
RHG29_HUMAN	Rho GTPase-activating protein 29 OS = Homo sapiens GN = ARHGAP29 PE = 1 SV = 2	141,974	3.3	37
ZFY27_HUMAN	Protrudin OS = Homo sapiens GN = ZFYVE27 PE = 1 SV = 1	45,814	3.4	35
RUVB1_HUMAN	RuvB-like 1 OS = Homo sapiens GN = RUVBL1 PE = 1 SV = 1	50,196	5	33
BLMH_HUMAN	Bleomycin hydrolase OS = Homo sapiens GN = BLMH PE = 1 SV = 1	52,528	2.4	33
TIAM2_HUMAN	T-lymphoma invasion and metastasis-inducing protein 2 OS = Homo sapiens GN = TIAM2 PE = 2 SV = 4	189,985	0.9	27
GON4L_HUMAN	GON-4-like protein OS = Homo sapiens GN = GON4L PE = 1 SV = 1	248,465	2.3	26
OR6S1_HUMAN	Olfactory receptor 6S1 OS = Homo sapiens GN = OR6S1 PE = 3 SV = 2	36,103	2.4	25
PKRI1_HUMAN	PRKR-interacting protein 1 OS = Homo sapiens GN = PRKRIP1 PE = 1 SV = 1	20,984	6.5	25
FBXL7_HUMAN	F-box/LRR-repeat protein 7 OS = Homo sapiens GN = FBXL7 PE = 2 SV = 1	54,540	3.1	25
RINI_HUMAN	Ribonuclease inhibitor OS = Homo sapiens GN = RNH1 PE = 1 SV = 2	49,941	2.6	24
KI26B_HUMAN	Kinesin-like protein KIF26B OS = Homo sapiens GN = KIF26B PE = 2 SV = 1	223,744	1.8	23
HMGB3_HUMAN	High mobility group protein B3 OS = Homo sapiens GN = HMGB3 PE = 1 SV = 4	22,965	4	23
RNF32_HUMAN	RING finger protein 32 OS = Homo sapiens GN = RNF32 PE = 1 SV = 1	41,490	1.9	22
BARD1_HUMAN	BRCA1-associated RING domain protein 1 OS = Homo sapiens GN = BARD1 PE = 1 SV = 2	86,593	3.3	21
TC1D4_HUMAN	Tctex1 domain-containing protein 4 OS = Homo sapiens GN = TCTEX1D4 PE = 1 SV = 1	23,338	3.2	21
PRA10_HUMAN	PRAME family member 10 OS = Homo sapiens GN = PRAMEF10 PE = 2 SV = 4	55,175	4	21
GTR7_HUMAN	Solute carrier family 2, facilitated glucose transporter member 7 OS = Homo sapiens GN = SLC2A7 PE = 2 SV = 2	55,692	1.6	20
